# The change in epidemiological characteristics and utilization of health services for the diagnosis and treatment of colorectal cancer: A hospital-based retrospective survey

**DOI:** 10.1016/j.clinsp.2026.101075

**Published:** 2026-09-02

**Authors:** Wenjie Liang, Haiyan Lu, Kaiyong Huang, Hongyuan Huang, Li Yang

**Affiliations:** a Department of Social Medicine, School of Public Health, Guangxi Medical University, Nanning, Guangxi, 530021, China; b Department of Occupational and Environmental Health, School of Public Health, Guangxi Medical University, Nanning, Guangxi, 530021, China

**Keywords:** Colorectal cancer, Epidemiological characteristics, Health service utilization, Changing trends

## Abstract

**Background:**

The incidence, mortality, epidemiological characteristics, and health utilization for the diagnosis and treatment of colorectal cancer in China have shown an upward trend over time, with substantial nationwide regional variation. We aimed to observe the shift in demographic and clinical characteristics and medical utilization for the diagnosis and treatment of colorectal cancer in Guangxi from 2005 to 2014.

**Methods:**

This study was a hospital-based retrospective survey. We enrolled patients with primary colorectal cancer in a tertiary A specialized cancer hospital diagnosed between 2005 and 2014. Patients' data were collected by trained investigators via the electronic hospital medical records. All data were calculated and analyzed using the Statistical Package for the Social Sciences version 20 statistical software.

**Results:**

Data of 1065 primary colorectal cancer patients were collected. The mean age at diagnosis was 57.33-years, and the male-to-female ratio was 1.51. The proportion of overweight/obese patients ranged from 4.95% to 23.76% between 2005 and 2014, with an upward trend. The percentage of patients with tobacco consumption increased from 4.00% to 25.45% between 2005 and 2014, and the proportion of patients undergoing CT increased from 12% to 90%; for MRI, from 0% to 25%; and for colonoscopy, from 18% to 74%. The positive rates of serum carcinoembryonic antigen with or without metastasis were 70.83% and 41.5% (p < 0.001). The positive rates of carcinoembryonic antigen and carbohydrate antigen 19-9 were positively correlated with stages at diagnosis (*r* = 0.31, 95% Confidence Interval: 0.18–0.34, p < 0.001) and (*r* = 0.35, 95% Confidence Interval: 0.21–0.47, p < 0.001), respectively.

**Conclusions:**

The rising percentage of overweight/obese and smokers among farmers poses challenges for the prevention, control, and treatment of colorectal cancer in Guangxi. The level of serum carcinoembryonic antigen before therapy can provide information for colorectal cancer with asymptomatic metastatic disease and advanced stage.

## Highlights

• Epidemiologic trends in colorectal cancer, Guangxi (2005–2014).
• Stage migration observed: Stage I–II up, Stage III down across years.
• Diagnostic shift: CT/MRI and colonoscopy up; X-Ray/ultrasound down.
• Pretherapy CEA and CA19-9 associated with metastasis and advanced stage.
• Increasing prevalence of overweight and smoking among patients.

## Introduction

The Global Cancer Observatory estimated in 2020 that more than 1.9 million new Colorectal Cancer (CRC) cases and 935,000 deaths occurred in 2020, and CRC ranked third in terms of incidence but second in terms of mortality.^1^ From 1990 to 2017, the age-standardized incidence rates of CRC increased globally, with substantial regional and national heterogeneity^2^ geographic disparity of diagnostic and treatment interval, cancer stages, and mortality in CRC patients was also observed.^3^ Furthermore, the incidence and risk of early-onset CRC (adolescent and young adult) continuously rose.^4^, ^5^, ^6^, ^7^, ^8^, ^9^, ^10^ Therefore, global trends of CRC incidence and mortality were grouped into three patterns: an increase in both incidence and mortality in the most recent decade; an increase in incidence with a concomitant decrease in mortality; and a decrease in both incidence and mortality.^11^

The Global Cancer Observatory calculated that 550,628 new cases and 283,751 deaths due to CRC occurred in 2020 in China, and CRC ranked second in terms of incidence and fifth in terms of mortality in all cancers.^12^ From 1990 to 2017, the incidence and mortality rates of CRC in China demonstrated increasing trends,^13^ with substantial regional variation nationwide.^13^^,^^14^ In Guangxi Zhuang Autonomous Region (Guangxi), the age-standardized incidence and mortality of CRC were ranked fourth and third in all cancers in 2016, respectively^14^; moreover, this showed declining trends in urban and rural populations from 2013 to 2016, with a greater decrease in rural populations than that in urban populations.^14^, ^15^, ^16^, ^17^

Despite the observed downward trend in the incidence and mortality of CRC in Guangxi, the number of new cases still increases due to the large population size. There is a constant transition to western dietary and lifestyle practices with economic growth, whereas the long-term shift in the epidemiology of the demographic and clinical characteristics of CRC in Guangxi was unclear. Although demographic and clinical characteristics of CRC for Guangxi have been previously reported,^18^^,^^19^ these are limited to fewer characteristics to analyze, and neglect the temporal trend, making an understanding of changes with time difficult.

This study aimed to demonstrate the shift in demographic and clinical characteristics of colorectal carcinoma and changes in healthcare utilization for CRC diagnosis and treatment in Guangxi from 2005 to 2014, providing evidence for future adjustments to prevention and control strategies.

## Materials and methods

### Study site and participants

This study was performed in a tertiary A specialized cancer hospital in Nanning, the capital city of Guangxi. The inclusion criteria were as follows: 1) Patients who were diagnosed with primary CRC and accepted the main therapy in the study hospital; 2) The first clinical visit/admission for CRC diagnosis and/or treatment occurred between January 1, 2005, and December 31, 2014; and 3) The availability of comprehensive demographic and clinical information, particularly details on disease stages and pathological diagnoses. Patients who only visited the study hospital for diagnosis, post-treatment follow-up, or outpatient radiotherapy were excluded. All data were obtained from the electronic medical record system.^20^

### Data acquisition and quality control

In this study, at least 100 patients were selected by the sampling method yearly from 2005 to 2014. We examined the date of the first visit/admission of patients in the study hospital. We then selected the first patients diagnosed in May 2005, June 2006, July 2007, August 2008, September 2009, October 2010, November 2011, December 2012, March 2013, and April 2014, following which we included cases until 100 eligible patients were obtained. We used a simplified table to filter patients with eligible and noneligible CRC. Colorectal cancer staging was determined according to the American Joint Committee on Cancer (AJCC) TNM classification, 7th edition.^20^ A survey was conducted on patients with primary CRC via a structured case report form offered by the Chinese National Cancer Center (CNCC). The following data were collected: 1) Demographic characteristics including sex, age, height, weight, tobacco and alcohol consumption statuses; 2) Clinical characteristics, such as clinical stage, pathological diagnosis, tumor differentiation, and metastatic state; 3) Technologies used for diagnosis and treatment data and; 4) Results of tumor markers.

Managers of our team attended a training course organized by the CNCC in Beijing. Several postgraduate students from the school of public health in Guangxi medical university were recruited, and then team managers trained them until there was a need for an examination. One investigator gathered data from the electronic medical record system of the study hospital and recorded it on the paper-based case report form, while another rechecked. Data were double-entered into the CNCC’s purpose-built database (Epidata 3.1, Odense, Denmark) and then submitted to the CNCC, after which they were supplemented or modified when needed.

### The implementation of tumor biomarkers test

The test was conducted daily by the laboratory department of the study hospital since the results were utilized by clinicians to guide them on diagnosis and treatment. We captured the serum level of Carbohydrate Antigen 19-9 (CA19-9) and Carcinoembryonic Antigen (CEA) and the corresponding normal reference values from the electronic medical record system; values that exceeded the corresponding upper limit of the normal threshold were considered positive, and those lower were considered negative.

### Statistical analysis

According to the official government reform plan, health reforms in China can be stratified into two phases: Phase One (2009 to 2011) and Phase Two (2012 onward).^21^ Hence, the study period fell within three calendar periods: 2005–2008; 2009–2011; and 2012–2014.

All data were analyzed using the Statistical Package for the Social Sciences version 20 statistical software (IBM Corp., Armonk, NY, USA). Demographic and clinical features were described using proportions; age at diagnosis was described by means; categorical variables were analyzed with either a chi-square test or Fisher exact test probability method when appropriate. The time trend of age at diagnosis from 2005 to 2014 was determined using Spearman correlation analysis. Temporal trends of proportion were determined using linear-by-linear association of the Chi-Square test. The influencing factors of the positive rate of CEA and CA19-9 were determined using the binary logistic regression analysis. Missing/unknown values were omitted from the statistical analyses. All tests of statistical significance were two-sided, and a p-value < 0.05 was considered statistically significant.

## Results

### Demographics and behavior characteristics

#### Overall demographics and behavior characteristics

Records of 1065 patients diagnosed with primary CRC from 2005 to 2014 were collected. The overall mean age at diagnosis was 57.33-years, and the total male-to-female ratio was 1.51. The majority of the patients were farmers and retirees (33.59% and 23.04%, respectively), and 44.49% had no insurance. The percentage of patients who consumed alcohol and tobacco was 9.18% and 6.68%, respectively. These data are provided in Table 1.

**Table 1:** Demographic and clinical characteristics of colorectal cancer in Guangxi during 2005‒2014.

Characteristics	Number of patients (%)				p‡
	2005‒2014	2005‒2011	2009‒2011	2012‒2014	
Age at diagnosis (year), Mean ± SD	57.33 ± 13.39	56.45 ± 13.71	58.74 ± 13.60	57.03 ± 12.65	0.041
Age at diagnosis (year), male, Mean ± SD	57.44 ± 13.56	56.41 ± 14.02	59.35 ± 13.69	57.02 ± 12.67	0.049
Age at diagnosis (year), female, Mean ± SD	57.14 ± 13.14	56.50 ± 13.23	57.91 ± 13.50	57.03 ± 12.67	0.570
Age group at diagnosis, Year					0.075
<45	220 (20.66)	92 (22.17)	66 (20.00)	62 (19.38)	
45‒59	336 (31.55)	130 (31.33)	89 (26.97)	117 (36.56)	
≥60	509 (47.79)	193 (46.51)	175 (53.03)	141 (44.06)	
Sex					0.475
Male	641 (60.19)	257 (61.93)	190 (57.58)	194 (60.62)	
Female	424 (39.81)	158 (38.07)	140 (42.42)	126 (39.38)	
Body mass index, kg/m^2^*					0.008
<18.5	179 (16.85)	84 (20.24)	54 (16.36)	41 (12.81)	b
18.5‒23.9	724 (68.17)	286 (68.92)	219 (66.36)	222 (69.38)	
≥24.0	159 (14.98)	45 (10.84)	57 (17.27)	57 (17.81)	a^,^^b^
Occupation*					0.001
Public servant	108 (10.45)	60 (15.04)	29 (8.92)	19 (6.15)	a^,^^b^
Manufacturing staff	112 (10.84)	50 (12.53)	26 (8.00)	36 (11.65)	
Farmer	347 (33.59)	107 (26.82)	125 (38.46)	115 (37.22)	a^,^^b^
Retiree	238 (23.04)	91 (22.81)	84 (25.85)	63 (20.39)	
Other	228 (22.07)	91 (22.81)	61 (18.77)	76 (24.60)	
Type of health insurance*					<0.001
Urban employee basic medical insurance	226 (21.48)	61 (14.84)	74 (22.63)	91 (28.98)	a^,^^b^
New rural cooperative medical scheme	254 (24.14)	6 (1.46)	113 (34.56)	135 (42.99)	a^,^^b^
No insurance	468 (44.49)	314 (76.40)	90 (27.52)	64 (20.38)	a^,^^b^
Others	104 (9.89)	30 (7.30)	50 (15.29)	24 (7.64)	
Alcohol consumption status*					<0.001
No	960 (90.82)	395 (95.18)	286 (86.67)	279 (89.42)	a^,^^b^
Yes	97 (9.18)	20 (4.82)	44 (13.33)	33 (10.58)	a^,^^b^
Tobacco consumption status*					<0.001
No	884 (83.32)	376 (90.82)	259 (78.96)	249 (78.06)	a^,^^b^
Yes	177 (16.68)	38 (9.18)	69 (21.04)	70 (21.94)	a^,^^b^
History of any CRC-related disease					0.918
Yes	55 (5.16)	20 (4.82)	18 (5.45)	17 (5.31)	
No	1010 (94.84)	395 (95.18)	312 (94.55)	303 (94.69)	
History of tumors					0.567
Yes	15 (1.41)	6 (1.45)	3 (0.91)	6 (1.88)	
No	1050 (98.59)	409 (98.55)	327 (99.09)	314 (98.13)	
Family history of tumors					<0.001
Yes	53 (4.98)	17 (4.10)	7 (2.12)	29 (9.06)	b^,^^c^
No	1012 (95.02)	398 (95.90)	323 (97.88)	291 (90.94)	b^,^^c^
Metastasis at diagnosis					0.179
No	762 (73.27)	288 (71.11)	235 (72.31)	239 (77.10)	
Yes	278 (26.73)	117 (28.89)	90 (27.69)	71 (22.90)	
Histologic type					0.896
Adenocarcinoma	988 (92.77)	384 (92.53)	309 (93.64)	295 (92.19)	
Mucinous adenocarcinoma	54 (5.07)	20 (4.82)	16 (4.85)	18 (5.63)	
Signet-ring cell carcinoma	6 (0.56)	2 (0.48)	2 (0.61)	2 (0.63)	
Other types	17 (1.60)	9 (2.16)	3 (0.91)	5 (1.56)	
Tumor differentiation*					<0.001
Well-differentiated	50 (6.93)	19 (9.60)	7 (2.79)	24 (8.82)	a^,^c
Moderately-differentiated	548 (76.01)	141 (71.21)	186 (74.10)	221 (81.25)	b
Poorly differentiated	123 (17.06)	38 (19.19)	58 (23.11)	27 (9.93)	b^,^^c^
Stage at diagnosis*					0.287
Stage Ⅰ	45 (4.48)	11 (2.72)	16 (5.13)	18 (6.25)	
Stage Ⅱ	321 (31.97)	122 (30.20)	103 (33.01)	96 (33.33)	
Stage Ⅲ	346 (34.46)	150 (37.13)	103 (33.01)	93 (32.29)	
Stage Ⅳ	292 (29.08)	121 (29.95)	90 (28.85)	81 (28.13)	
Tumor location*					0.810
Right colon	256 (24.06)	101 (24.34)	84 (25.45)	71 (22.26)	
Left colon	250 (23.50)	98 (23.61)	80 (24.24)	72 (22.57)	
Rectum	542 (50.94)	209 (50.36)	160 (48.48)	173 (54.23)	
Colon and rectum	16 (1.50)	7 (1.69)	6 (1.82)	3 (0.94)	

CRC, Colorectal Cancer; SD, Standard Deviation.

Notes:

⁎ The number of patients with not report included 3 patients with unknown body mass index, 32 patients with unknown occupation, 13 patients with unknown types of health insurance, 8 patients with unknown alcohol consumption status, 4 patients with unknown tobacco consumption status, 267 patients with unknown tumor differentiation, 61 patients with unknown stage at diagnosis, and 1 patient with unknown tumor location.

‡ Comparisons were performed among 2005‒2008, 2009‒2011, 2012‒2014.

a 2005‒2008 vs. 2009‒2011, p < 0.05.

b 2005‒2008 vs. 2012‒2014, p < 0.05.

c 2009‒2011 vs. 2012‒2014, p < 0.05. ^†^CRC-related diseases included colorectal polyps, inflammatory bowel disease, and other benign colorectal conditions.

### Time trends in demographics and behavior characteristics

**Fig. 1:**
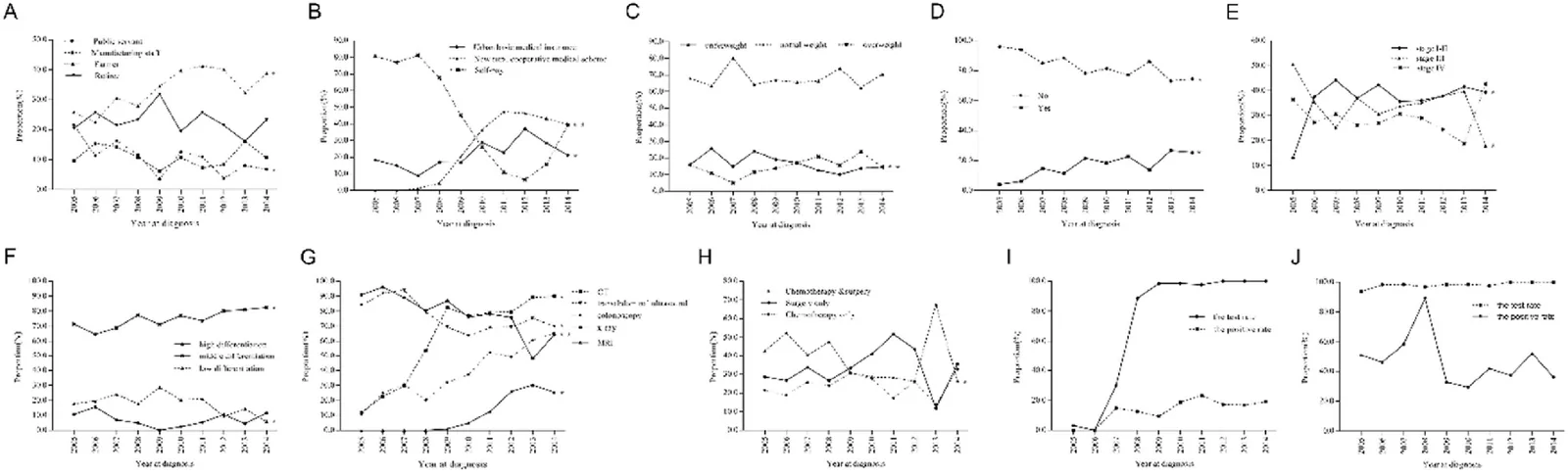
Temporal trends in demographic, behavioral, clinical, and diagnostic characteristics of colorectal cancer patients in Guangxi, 2005–2014. (A) Time trends of patients in different occupations; (B) Time trends of patients covered by different medical insurances; (C) Time trends of patients with different body mass index categories (underweight, normal weight, overweight/obese); (D) Time trend of patients who are smokers or non-smokers; (E) Time trend of patients with different cancer stages (Stage I/II, Stage III, Stage IV); (F) Time trend of patients with different tumor differentiation levels (well/moderately differentiated, poorly differentiated); (G) Time trends of patients undergoing different diagnostic tests (X-Ray, CT, MRI, transabdominal ultrasound, colonoscopy); (H) Time trends of patients treated by different modalities (surgery alone, surgery plus chemotherapy, chemotherapy alone, others); (I) Time trend of the proportion of patients with positive serum CEA before treatment; (J) Time trend of the proportion of patients with positive serum CA19-9 before treatment. Abbreviations: CT, computed tomography; MRI, magnetic resonance imaging; CEA, carcinoembryonic antigen; CA19-9, carbohydrate antigen 19-9. Panels are labeled A–J in the top-left corner of each corresponding image.

The percentage of farmers increased from 25.81% to 33.59% between 2005 and 2014 (p-trend = 0.001). The percentage of patients with a new rural cooperative medical scheme and those with urban employee basic medical insurance increased from 2005 to 2014 (all p-trend < 0.001), while that of patients with no insurance decreased from 80.61% to 39.42% between 2005 and 2014 (p-trend < 0.001). The proportion of overweight or obese patients ranged from 4.95% to 23.76% between 2005 and 2014, with an upward trend (p-trend = 0.011). The percentage of patients with tobacco consumption increased from 4.00% to 25.45% between 2005 and 2014 (p-trend < 0.001). Quantitatively, the annual odds of being overweight/obese increased by 18% (OR = 1.18, 95% CI: 1.04–1.34), and the annual odds of tobacco consumption increased by 22% (OR = 1.22, 95% CI: 1.13–1.32). These results are illustrated in Fig. 1.

### Clinical and pathological characteristics

#### Overall clinical and pathological characteristics

In total, 972 patients reported clinical symptoms. The main clinical symptoms were occult or overt rectal bleeding (27.88%), weight loss (27.37%), abdominal distension (8.95%), diarrhea (6.38%), increasing stool frequency (5.25%), constipation (4.01%), and 26.73% of patients were diagnosed with metastatic disease. The most common histologic type was adenocarcinoma (92.77%); 76.01% of the patients with adenocarcinoma were moderately differentiated, and 63.54% were diagnosed at an advanced stage (stages Ⅲ and Ⅳ). Further information is listed in Table 1.

#### Time trends in clinical and pathological characteristics

The percentage of patients diagnosed with stage Ⅰ and stage Ⅱ CRC increased from 13.13% to 39.33% between 2005 and 2014 (p-trend = 0.011), while that of those diagnosed with stage Ⅲ cancer decreased from 50.51% to 17.98% between 2005 and 2014 (p-trend = 0.027). The percentage of patients with poorly differentiated tumors ranged from 17.86% to 5.81%, showing a downward trend from 2005 to 2014 (p-trend = 0.001). This information is presented in Fig. 1.

### Medical service utilization

#### Overall medical service utilization

According to the utilization of CRC-related diagnostic techniques from 2005 to 2014, chest X-Ray was the most commonly used technology (78.59%), followed by transabdominal ultrasound (76.43%) and Computed Tomography (CT) scanning (62.44%). The proportion of patients who conducted the CA19-9 and CEA tests were 71.68% and 97.08%, respectively. The proportion of patients undergoing pretherapeutic serum CEA and CA19-9 testing increased during 2005–2008 and then stabilized during 2009–2014. According to treatment regimens, surgery combined with chemotherapy was the most common treatment option (38.83%). Further details are provided in Table 2.

**Table 2:** The usage of service for diagnosis and treatment of colorectal cancer in Guangxi during 2005‒2014.

Characteristics	Number of patients, n (%)				p*
	2005‒2014	2005‒2008	2009‒2011	2012‒2014	
Utilization of diagnostic technique					
X-Ray	837 (78.59)	369 (88.92)	266 (80.61)	202 (63.13)	<0.001^a^^,^^b^^,^^c^
CT	665 (62.44)	126 (30.36)	263 (79.70)	276 (86.25)	<0.001^a^^,^^b^
MRI	107 (10.06)	0.00 (0.00)	20 (6.06)	87 (27.19)	<0.001^a^^,^^b^^,^^c^
Transabdominal ultrasound	814 (76.43)	362 (87.23)	223 (67.58)	229 (71.56)	<0.001^a^^,^^b^
Colonoscopy	411 (38.63)	90 (21.74)	134 (40.61)	187 (58.44)	<0.001^a^^,^^b^^,^^c^
Utilization of cancer biomarkers test					
Carbohydrate antigen19-9 test	762 (71.68)	130 (33.33)	315 (95.45)	317 (99.69)	<0.001^a^^,^^b^^,^^c^
Cancer embryo antigen test	1032 (97.08)	396 (95.42)	319 (96.67)	317 (99.69)	0.003^b^^,^^c^
Receipt of treatment					<0.001
Surgery alone	355 (33.46)	119 (28.81)	138 (41.95)	98 (30.72)	a^,^^c^
Surgery and chemotherapy	412 (38.83)	190 (46.00)	96 (29.18)	126 (39.50)	a^,^^c^
Chemotherapy alone	254 (23.94)	93 (22.52)	83 (25.23)	78 (24.45)	
Others	40 (3.77)	11 (2.66)	12 (3.65)	17 (5.33)	

CT, Computed Tomography; MRI, Magnetic Resonance Imaging.

Notes: * Comparisons were performed among 2005‒2008, 2009‒2011, 2012‒2014.

a 2005‒2008 vs. 2009‒2011, p < 0.05.

b 2005‒2008 vs. 2012‒2014, p < 0.05.

c 2009‒2011 vs. 2012‒2014, p < 0.05.

#### Time trends in medical service utilization

The rate of utilization of chest X-Ray and transabdominal ultrasound decreased from 91.00% to 64.55% and 84.00% to 70.00% between 2005 and 2014 (p-trend < 0.001), (p-trend < 0.001), respectively. In contrast, the rate of utilization of CT, magnetic resonance imaging (MRI), and colonoscopy increased from 12.00% to 90.00%, 0.00% to 25.45%, and 18.00% to 73.64% between 2005 and 2014, respectively, (p-trend < 0.001), (p-trend < 0.001) (p-trend < 0.001). The percentage of surgery combined with chemotherapy decreased from 42.86% to 26.36% between 2005 and 2014 (p-trend = 0.032). This information is displayed in Fig. 1.

### CA19-9 and CEA

#### The positive rate of CA19-9 and CEA

The positive rates of serum CEA and CA19-9 were computed in patients who did not receive treatment in other hospitals, and the serum levels of CEA and CA19-9 before treatment were detected in the study hospital. There were notable differences in the positive rates of serum CEA and CA19-9 before treatment in patients with or without metastasis and disease stages at diagnosis in the three calendar periods. Additional information is presented in Table 3, Table 4.

**Table 3:** The positive rate of CEA by demographic and clinical characteristics of colorectal cancer in Guangxi during 2005‒2014.

Characteristics	2005‒2014			2005‒2008			2009‒2011			2012‒2014		
	N° of patients	Positive rate, n (%)	p-value	N° of patients	Positive rate, n (%)	p-value	N° of patients	Positive rate, n (%)	p-value	N° of patients	Positive rate, n (%)	p-value
Total	663	308 (46.46)		224	138 (61.61)		211	74 (35.07)		228	96 (42.11)	
Sex			0.700			0.240			0.009			0.896
Male	397	182 (45.84)		141	91 (64.54)		117	32 (27.35)		139	59 (42.45)	
Female	266	126 (47.37)		83	47 (56.63)		94	42 (44.68)		89	37 (41.57)	
Age group at diagnosis, year			0.520			0.103			0.609			0.296
<45	122	51 (41.8)		42	20 (47.62)		39	11 (28.21)		41	20 (48.78)	
45‒59	203	96 (47.29)		73	49 (67.12)		46	17 (36.96)		84	30 (35.71)	
≥60	338	161 (47.63)		109	69 (63.30)		126	46 (36.51)		103	46 (44.66)	
Body mass index, kg/m^2^			0.096			0.687			0.287			0.460
<18.5	85	45 (52.94)		29	19 (65.52)		28	12 (42.86)		28	14 (50.00)	
18.5‒23.9	470	222 (47.23)		173	104 (60.12)		137	50 (36.50)		160	68 (42.50)	
≥24.0	108	41 (37.96)		22	15 (68.18)		46	12 (26.09)		40	14 (35.00)	
Metastasis at diagnosis			**<0.001**			**0.009**			**<0.001**			**<0.001**
No	531	218 (41.05)		178	103 (57.87)		169	49 (28.99)		184	66 (35.87)	
Yes	120	85 (70.83)		40	32 (80.00)		39	24 (61.54)		41	29 (70.73)	
Tobacco consumption status			0.284			0.823			0.203			0.481
No	543	257 (47.33)		201	123 (61.19)		162	60 (37.04)		180	74 (41.11)	
Yes	117	49 (41.88)		22	14 (63.64)		48	13 (27.08)		47	22 (46.81)	
Alcohol consumption status			0.277			1.000			0.363			0.121
No	587	275 (46.85)		209	125 (59.81)	0.039	182	66 (36.26)		196	84 (42.86)	
Yes	70	28 (40.)		15	13 (86.67)		29	8 (27.59)		26	7 (26.92)	
Stage at diagnosis			**<0.001**			0.071			**<0.001**			**<0.001**
Stage Ⅰ and Ⅱ	265	94 (35.47)		79	44 (55.70)		91	24 (26.37)		95	26 (27.37)	
Stage Ⅲ	231	118 (51.08)		95	60 (63.16)		71	25 (35.21)		65	33 (50.77)	
Stage Ⅳ	128	87 (67.97)		43	33 (76.74)		38	24 (63.16)		47	30 (63.83)	
Tumor location			**0.022**			0.113			**0.004**			0.108
Right colon	143	58 (40.56)		45	31 (68.89)		50	13 (26.00)		48	14 (29.17)	
Left colon	147	82 (55.78)		54	37 (68.52)		52	28 (53.85)		41	17 (41.46)	
Rectum	363	162 (44.63)		122	67 (54.92)		106	32 (30.19)		135	63 (46.67)	
Histologic type			0.994			0.118			0.911			0.235
Non-adenocarcinoma	43	20 (46.51)		18	8 (44.44)		9	3 (33.33)		16	9 (56.25)	
Adenocarcinoma	620	288 (46.45)		206	130 (63.11)		202	71 (35.15)		212	87 (41.04)	
Degree of differentiation			0.171			0.675			0.625			0.120
Well and moderately	398	173 (43.47)		76	51 (67.11)		137	49 (35.77)		185	73 (39.46)	
Poorly	77	40 (51.95)		18	13 (72.22)		40	16 (40.00)		19	11 (57.89)	

CEA, Cancer Embryo Antigen; N°, Number.

**Table 4:** The positive rate of CA19-9 by demographic and clinical characteristics of colorectal cancer in Guangxi during 2005‒2014.

Characteristics	2005‒2014			2005‒2008			2009‒2011			2012‒2014		
	N° of patients	Positive rate, n (%)	p-value	N° of patients	Positive rate, n (%)	p-value	N° of patients	Positive rate, n (%)	p-value	N° of patients	Positive rate, n (%)	p-value
Total	482	89 (18.46)	NA	52	10 (16.13)	NA	205	38 (18.54)		215	41 (19.07)	NA
Sex			**0.044**			0.123			0.909			0.056
Male	284	44 (15.49)		41	4 (9.76)		115	21 (18.26)		128	19 (14.84)	
Female	198	45 (22.73)		21	6 (28.57)		90	17 (18.89)		87	22 (25.29)	
Age group at diagnosis, year			0.749			1.000			0.755			0.831
<45	84	16 (19.05)		12	2 (16.67)		34	7 (20.59)		38	7 (18.42)	
45‒59	146	24 (16.44)		18	3 (16.67)		47	7 (14.89)		81	14 (17.28)	
≥60	252	49 (19.44)		32	5 (15.63)		124	24 (19.35)		96	20 (20.83)	
Body mass index, kg/m^2^			0.548			0.401			0.959			0.381
<18.5	60	8 (13.33)		8	1 (12.50)		28	5 (17.86)		24	2 (8.33)	
18.5‒23.9	327	63 (19.27)		45	9 (20.00)		132	24 (18.18)		150	30 (20.00)	
≥24.0	95	18 (18.95)		0	0 (0.00)		45	9 (20.00)		41	9 (21.95)	
Metastasis at diagnosis			**<0.001**			**0.038**			**<0.001**			**<0.001**
No	395	46 (11.65)		48	5 (10.42)		166	19 (11.45)		181	22 (12.15)	
Yes	82	41 (50.00)		14	5 (35.71)		37	18 (48.65)		31	18 (58.06)	
Tobacco consumption status			0.072			0.586			0.468			0.140
No	379	75 (19.79)		55	10 (18.18)		154	29 (18.83)		170	36 (21.18)	
Yes	100	12 (12.00)		7	0 (0.00)		49	7 (14.29)		44	5 (11.36)	
Alcohol consumption status			**0.033**			1.000			0.533			**0.010**
No	416	82 (19.71)		54	9 (16.67)		177	34 (19.21)		185	39 (21.08)	
Yes	60	5 (8.33)		8	1 (12.5)		28	4 (14.29)		26	0 (0.00)	
Stage at diagnosis			**<0.001**			0.051			**<0.001**			**<0.001**
Stage Ⅰ and Ⅱ	210	20 (9.52)		26	2 (7.69)		90	9 (10.00)		94	9 (9.57)	
Stage Ⅲ	155	23 (14.84)		23	3 (13.04)		68	8 (11.76)		64	12 (18.75)	
Stage Ⅳ	85	42 (49.41)		13	5 (38.46)		36	18 (50.00)		75	19 (52.78)	
Tumor location			0.057			**0.015**			0.427			0.714
Left colon	103	27 (26.21)		16	6 (37.5)		49	12 (24.49)		38	9 (23.68)	
Right colon	110	16 (14.55)		16	0 (0.00)		49	8 (16.33)		45	8 (17.78)	
Rectum	262	44 (16.79)		29	4 (13.79)		105	17 (16.19)		128	23 (17.97)	
Histologic type			0.972			1.000			0.234			0.240
Non‒adenocarcinoma	22	4 (18.18)		1	0 (0.00)		7	3 (42.86)		14	1 (7.14)	
Adenocarcinoma	460	85 (18.48)		61	10 (16.39)		198	35 (17.68)		201	40 (19.9)	
Degree of differentiation			0.140			0.591			0.725			0.064
Well and moderately	340	62 (18.24)		29	4 (13.79)		135	25 (18.52)		176	33 (18.75)	
Poorly	65	17 (26.15)		8	2 (25.00)		38	8 (21.05)		19	7 (36.84)	

CA19-9, Carbohydrate Antigen19-9; N°, Number.

The positive rate of CEA combined with CA19-9 was different in the presence or absence of metastasis and disease stage at diagnosis; detailed data are provided in Table 5. The binary logistic regression models showed that metastasis and the disease stage at diagnosis can affect the serum CEA level, and that metastasis and sex can influence the expression of serum CA19-9 (Table 6). Notably, the OR for metastasis in the CEA model had a wide 95% CI (1.02–17.53), suggesting imprecision likely due to the relatively small number of metastatic cases with complete data and correlation among predictors; therefore, this estimate should be interpreted cautiously.

**Table 5:** The positive rate of CEA combined with CA19-9 by demographic and clinical characteristics of colorectal cancer.

Characteristics	2005‒2014			2005‒2008			2009‒2011			2012‒2014		
	N° of patients	Positive rate, n (%)	p-value	N° of patients	Positive rate, n (%)	p-value	N° of patients	Positive rate, n (%)	p-value	N° of patients	Positive rate, n (%)	p-value
Total	516	256 (49.61)		73	58 (79.45)		213	91 (42.72)		230	107 (46.52)	
Sex			**0.034**			0.654			**0.001**			0.972
Male	304	139 (45.72)		45	35 (77.78)		119	39 (32.77)		140	65 (46.43)	
Female	212	117 (55.19)		28	23 (82.14)		94	52 (55.32)		90	42 (46.67)	
Age group at diagnosis, year			0.708			0.130			0.729			0.617
<45	96	45 (46.88)		15	9 (60.00)		39	15 (38.46)		42	21 (50.)	
45‒59	155	75 (48.39)		23	20 (86.96)		47	19 (40.43)		85	36 (42.35)	
≥60	265	136 (51.32)		35	29 (82.86)		127	57 (44.88)		103	50 (48.54)	
Body mass index, kg/m^2^			0.106			0.177			0.417			0.745
<18.5	66	38 (57.58)		10	9 (90.00)		28	15 (53.57)		28	14 (50.)	
18.5‒23.9	353	178 (50.42)		54	44 (81.48)		28	15 (53.57)		161	76 (47.2)	
≥24.0	97	40 (41.24)		9	5 (55.56)		138	58 (42.03)		41	17 (41.46)	
Metastasis at diagnosis			**<0.001**			1.000			**<0.001**			**<0.001**
No	411	177 (43.07)		57	45 (78.95)		169	61 (36.09)		185	71 (38.38)	
Yes	99	77 (77.78)		16	13 (81.25)		41	29 (70.73)		42	35 (83.33)	
Tobacco consumption status			0.186			0.356			0.061			0.733
No	410	209 (50.98)		66	51 (77.27)		162	74 (45.68)		182	84 (46.15)	
Yes	103	45 (43.69)		7	7 (100.00)		49	15 (30.61)		47	23 (48.94)	
Alcohol consumption status			0.059			0.289			0.171			**0.043**
No	447	227 (50.78)		65	50 (76.92)		184	82 (44.57)		198	95 (47.98)	
Yes	63	24 (38.1)		8	8 (100.00)		29	9 (31.03)		26	7 (26.92)	
Stage at diagnosis			**<0.001**			1.000			**<0.001**			**<0.001**
Stage Ⅰ and Ⅱ	215	82 (38.14)		28	23 (82.14)		91	31 (34.07)		96	28 (29.17)	
Stage Ⅲ	164	86 (52.44)		28	23 (82.14)		71	28 (39.44)		65	35 (53.85)	
Stage Ⅳ	103	78 (75.73)		15	12 (80.00)		40	29 (72.5)		48	37 (77.08)	
Tumor location			**0.043**			0.807			**0.002**			0.139
Left colon	113	65 (57.52)		19	14 (73.68)		52	33 (63.46)		42	18 (42.86)	
Right colon	117	48 (41.03)		17	14 (82.35)		52	17 (32.69)		48	17 (35.42)	
Rectum	277	138 (49.82)		35	28 (80.00)		106	40 (37.74)		136	70 (51.47)	
Histologic type			0.526			0.371			0.652			0.419
Non-adenocarcinoma	27	15 (55.56)		2	1 (50.00)		9	5 (55.56)		16	9 (56.25)	
Adenocarcinoma	489	241 (49.28)		71	57 (80.28)		204	86 (42.16)		214	98 (45.79)	
Degree of differentiation			0.277			1.000			0.746			0.118
Well and moderately	360	170 (47.22)		35	27 (77.14)		138	60 (43.48)		187	83 (44.39)	
Poorly	68	37 (54.41)		8	6 (75.00)		41	19 (46.34)		19	12 (63.16)	

CEA, Cancer Embryo Antigen; CA19-9, Carbohydrate Antigen19-9; N°, Number.

p-values of “1.000″ indicate exact values from Fisher's exact test (due to small expected cell counts).

**Table 6:** Binary logistic regression analysis of tumor biomarkers and demographic and clinical characteristics for colorectal cancer.

Parameter	β	p-value	OR (95% CI)
**CEA**			
Intercept	−0.66	<0.001	0.52
Metastasis at diagnosis (Yes vs. No)	1.44	0.047	4.23 (1.02‒17.53)
Stage at diagnosis (Ⅲ vs. ⅠandⅡ)	0.64	0.001	1.90 (1.31‒2.75)
Stage at diagnosis (Ⅳ vs. ⅠandⅡ)	−0.01	0.988	0.99 (0.24‒4.06)
**CA19-9**			
Intercept	−2.41	<0.001	0.09
Sex (Female vs. Male)	0.69	0.012	2.00 (1.16‒3.45)
Metastasis at diagnosis (Yes vs. No)	2.08	<0.001	7.99 (4.51‒14.14)
**CEA combined with CA19-9**			
Intercept	−0.71	<0.001	0.49
Sex (Female vs. Male)	0.50	0.012	1.66 (1.12‒2.45)
Stage at diagnosis (Ⅲ vs. ⅠandⅡ)	0.55	0.010	1.74 (1.14‒2.66)
Stage at diagnosis (Ⅳ vs. ⅠandⅡ)	1.73	<0.001	5.62 (3.22‒9.83)

CEA, Cancer Embryo Antigen; CA19-9, Carbohydrate Antigen19-9.

The correlation between the positive rate of CEA, CA19-9, and disease stage at diagnosis To confirm that serum levels of CEA and CA19-9 before treatment were associated with staging, we further assessed the correlations between the positive rate of biomarkers and stages at diagnosis from 2009 to 2014. The positive rate of CEA was positively correlated with stages at diagnosis (*r* = 0.31, 95% Confidence Interval [95% CI]: 0.18–0.34, p < 0.001). Similarly, the positive rate of CA19-9 was also positively correlated to stages at diagnosis (*r* = 0.35, 95% CI: 0.21–0.47, p < 0.001), and the positive rate of CEA combined with CA19-9 was positively correlated with stages at diagnosis (*r* = 0.38, 95% CI: 0.25–0.51, p < 0.001).

#### Time trend in the detection and positive rates of CA19-9 and CEA expressions

No obvious shift of the positive rate of serum CA19-9 expression before treatment was observed between 2005 and 2014 (p-trend = 0.278). With regard to positivity, the positive rate of serum CEA before treatment showed a downward trend from 2005 to 2014, (p for trend = 0.003); stabilizing after 2009 (p-trend = 0.096). Additional information is presented in Fig. 1.

## Discussion

As part of the cancer screening program in urban China, this study is a significant high-quality, long-term retrospective epidemiology survey of CRC in Guangxi. We observed that demographics, behavior characteristics, and clinical features such as occupation, medical insurance status, body mass index, tobacco consumption, stages at diagnosis, and tumor differentiation had shifted over 10-years. The utilization of diagnosis techniques also changed remarkably, whereas treatment regimens showed no marked shift. Furthermore, the rate of tumor marker tests continually increased. However, the positive rate was kept stable and was associated with metastasis and the disease stage. In this study, we collected data on demographic and clinical characteristics and usage of medical services over 10-years for the diagnosis and treatments of CRC to comprehensively reveal the long-term dynamic temporal tendency. However, this was not similar to other studies.^18^^,^^19^

In the present study, the observed mean age at diagnosis was 57.33-years, which remained constant over 10-years; this is slightly above that (56-years) observed in a previous study that analyzed patients diagnosed with CRC between 1993 and 2007 in Guangxi,^18^ and was slightly below that (59-years) reported in another study that analyzed patients diagnosed with CRC between 2003 and 2011 in Guangxi.^19^ The average age at diagnosis in our study was below the average age at diagnosis reported in China (59.3-years)^20^ and the global average age at diagnosis (68.8-years).^22^ The male-to-female ratio (1.57) observed in this study was similar to that reported in two studies that analyzed patients with CRC in Guangxi (1.46 and 1.61).^18^^,^^19^ Furthermore, the ratio was higher than that (1.34) reported in a multicenter retrospective survey^20^ and that (1.37) reported in the 2017 China cancer registration annual report.^23^ These results indicate that CRC patients in Guangxi are diagnosed at a younger age and have a higher male-to-female ratio compared with national averages.

This study revealed that the proportion of patients with CRC in Guangxi who were underweight showed a downward trend while the percentage of patients who were overweight or obese showed an upward trend from 2005 to 2014. This change observed in the patients who were overweight or obese conformed with a national multicenter survey.^20^ Obesity has been confirmed as a risk factor for CRC.^24^, ^25^, ^26^ For several decades, there have been remarkable changes in the dietary structure of the Chinese population, characterized by the replacement of the classical diets by more westernized ones (cereals, low-fat mixed dishes, and decreased vegetable intake, while the intake of animal and processed foods, sugars, sweetened beverages, and ultra-processed foods high in energy, fat, sugar, and salt increased),^27^^,^^28^ leading to the prevalence of undernutrition and overnutrition (overweight or obesity) in China.^29^ This implies a gradual improvement in the standard of living and lifestyle changes in Guangxi over 10-years. However, it also reinforces the need to strengthen public awareness of the benefit of healthy diets and appropriate physical activity to prevent CRC.

In this study, the percentage of patients with CRC who were public servants demonstrated a declining trend, whereas that of farmers showed an increasing trend in Guangxi from 2005 to 2014. The change in the percentage of public servants with CRC may be attributed to improved awareness of the relationship between physical inactivity and the development of the disease, while the shift in the percentage of farmers may be due to the sedentary lifestyle in rural areas and the large number of farmers in China.

In addition, our study revealed that the proportion of patients with CRC without medical insurance showed a marked decline in Guangxi from 2005 to 2014. This is due to China’s healthcare reform, which has enhanced health insurance coverage to over 90% since 2010.^21^ Furthermore, China has made substantial progress in improving equal access to care and enhancing financial protection from 2009 to 2019, especially for people of a lower socioeconomic status.^30^, ^31^, ^32^

In this study, an increase was observed in the percentage of tobacco consumption of patients with CRC over time. CRC was designated to be causally related to smoking by the International Agency for Research on Cancer Monographs.^33^^,^^34^ Although smoking prevalence in China decreased from 30.1% in 2000 to 25.6% in 2016 and will continue to reduce to 20%, which is the goal of Healthy China 2030,^35^^,^^36^ following the shift pattern of the prevalence of smoking and cancer deaths in the USA, smoking-related cancer deaths are expected to decrease in 40-years.^37^ Therefore, there is an expected increase in the percentage of smoking patients with CRC in Guangxi in future studies.

Furthermore, the proportion of patients diagnosed with stage Ⅰ and Ⅱ CRC showed an upward trend, while the percentage diagnosed with stage Ⅲ declined from 2005 to 2014. This pattern may be mainly due to healthcare reform, which increased hospital admission and outpatient visit rates,^30^ and to improved socioeconomic status, which enhanced health awareness and promoted earlier cancer detection. The relatively younger mean age at diagnosis in Guangxi may reflect regional demographic and lifestyle characteristics, including a larger rural population, earlier exposure to risk factors such as tobacco and dietary transitions, and lower life expectancy compared with national and global levels, all of which may contribute to earlier onset of CRC.

The utilization of CRC-related diagnostic techniques in Guangxi varied remarkably from 2005 to 2014, indicating gradual improvement in CRC diagnosis and treatment. This trend was consistent with the overall change observed in China.^20^ Since accurate diagnosis is beneficial for conducting optimal treatment regimens, the application of CT and colonoscopy has gradually increased in CRC diagnosis before the first protocol for the diagnosis and treatment of CRC was made in China.^38^ In the latest Chinese protocol for the diagnosis and treatment of CRC (2020 edition), CT and MRI were ranked the first and second of all recommended examination methods, respectively.

The present survey found that the rate of the pretherapeutic serum CEA test and CA19-9 test in CRC patients grew first during 2005–2008 and then became stable during 2009–2014. This shift may be due to the recommendations of the domestic and foreign protocols about diagnosis and treatment for CRC. The American Society of Clinical Oncology and the European Group on Tumor Markers recommended that preoperative CEA testing should be used for staging/treatment planning and providing prognostic information for CRC patients^39^^,^^40^; nevertheless, the former did not recommend CA 19-9 for diagnosis, staging, surveillance, or monitoring treatment of patients with colorectal cancer,^39^ whereas the latter suggested that preoperative levels of CA 19-9 may provide independent prognostic information for patients with CRC. The first-edition Chinese protocol of diagnosis and treatment of colorectal cancer was established in 2010 and revised three times. The pretherapeutic serum CEA and CA19-9 testing were recommended in all four editions of the Chinese protocol of diagnosis and treatment of colorectal cancer.^38^^,^^41^, ^42^, ^43^ Our study discovered that a combination of the pretherapeutic serum CEA with CA 19-9 can detect more patients with metastatic disease and those diagnosed at advanced stage. This indicated that combination of the pretherapeutic serum CEA with CA19-9 may be helpful for identify asymptomatic patients with metastatic disease or advanced stage.

The total positive rate of the pretherapeutic serum levels of CEA and CA19-9 of CRC patients in Guangxi was similar to that reported by a study conducted in Jilin province in China.^44^ In this study, we observed that the positive rate of the pretherapeutic serum CA19-9 and CEA levels in CRC patients with metastatic disease at diagnosis was significantly higher than that of those without metastatic disease. We also observed that these rates increased with clinical stages. The relationship between the clinical stage and the tumor makers in this study was in accordance with previous studies.^44^, ^45^, ^46^ This indicated that CRC patients with an elevated pretherapeutic serum CA19-9 or CEA level should be further investigated to detect asymptomatic metastatic disease and advanced stage early.

This study had several limitations. First, the survey was conducted only in a tertiary A special tumor hospital in the capital city (Nanning) of Guangxi, which may have caused selection bias. Second, the specific test method and the test kit of tumor markers were not available because the study data were retrospectively collected several years after testing. Therefore, considering the possibility of using different test methods and test kits in different years, we only calculated the positive rate of tumor markers in place of the level of tumor markers. Third, the sample size was relatively small; as part of the cancer screening program in urban China, this study used a sampling method, which gathered only about 1000 primary CRC cases over 10-years, which may also have reduced the precision of some regression estimates (e.g., metastasis in the CEA model). Fourth, our sampling method selected patients from a single month per year, which may not fully represent seasonal variations in CRC diagnosis or healthcare seeking behavior. Lastly, the completeness and accuracy of patient information based on the electronic medical record system of the study hospital may be biased.

Despite the stated limitations, the findings of this study have significant and practical implications. Time trends observed in our study provide a critical perspective of understanding the change in the demographic and clinical characteristics of CRC and the change in the utilization of technology and service in the diagnosis and treatment of CRC in Guangxi. Furthermore, these results indicated the effect of prevention strategies, control, and management of CRC in the past and provided a significant baseline for assessing the measures of prevention and control of CRC in Guangxi.

## Conclusion

In conclusion, the increasing percentage of patients who are overweight/obese and smoke, as well as the increasing population of farmers, pose challenges for CRC prevention, control, and treatment in Guangxi. The utilization of CT, MRI, and colonoscopy for diagnosing CRC has significantly increased, and the serum level of CEA before therapy can provide information for diagnosing CRC with asymptomatic metastatic disease and advanced stage.

## Abbreviations

CRC, Colorectal Cancer; CanSPUC, Cancer Screening Program in Urban China; CRF, Case Report Form; CNCC, Chinese National Cancer Centre.

## Ethics approval and consent to participate

The survey was approved by the Institutional Review Board of the cancer hospital at the Chinese Academy of Medical Sciences as a component of a broader screening program (program 15-071/998, since the retrospective and noninterventional design of the survey, informed consent from participants were waived). All experimented and procedures were performed in accordance with the Declaration of Helsinki.

## Consent for publication

Not applicable.

## Data availability

Since the National Health and Family Planning Commission (currently National Health Commission) of China and the National Cancer Center of China have formulated security measures. The data are not publicly available due to national policy restrictions; however, de-identified data supporting the findings of this study are available from the corresponding author upon reasonable request for validation purposes, subject to institutional and national regulations.

## Authors’ contributions

Yang Li designed and supervised the work and contributed to the revision of the intellectual content. Liang Wenjie was responsible for the specific implementation of the project, collected and analyzed the data, and drafted the manuscript. Lu Haiyan mainly contributed to the analysis and interpretation of the data. Huang Kaiyong mainly analyzed the data and made some comments when revising the manuscript. Hongyuan Huang mainly analyzed data.

## Funding

Supported by the Guangxi Sub-project of the “National Major Public Health Service Project”; Funded by the Guangxi Natural Science Foundation Youth Fund Project (n° 2022GXNSFBA035641).

## Declaration of competing interest

The authors declare no conflicts of interest.

## Appendix Supplementary materials

Image, application 1

Image, application 2
