# Supplementary material for: The change in epidemiological characteristics and utilization of health services for the diagnosis and treatment of colorectal cancer: A hospital-based retrospective survey
Source: Clinics (Sao Paulo). 2026 Sep 2;81:101075. doi: 10.1016/j.clinsp.2026.101075 (PMC13570314; doi:10.1016/j.clinsp.2026.101075)
Supplement: Supplementary file 1 [file mmc1.docx]

**CLINICS-D-25-00580_Supplementary Material**

**Supplementary Table 1** Annual number of colorectal cancer patients included in the study (2005–2014).

| **Year** | **Nº of patients enrolled** |
| --- | --- |
| 2005 | 102 |
| 2006 | 106 |
| 2007 | 108 |
| 2008 | 110 |
| 2009 | 105 |
| 2010 | 107 |
| 2011 | 108 |
| 2012 | 107 |
| 2013 | 106 |
| 2014 | 106 |
| **Total** | **1,065** |
